# Metagenomic analysis of the complex microbial consortium associated with cultures of the oil‐rich alga *Botryococcus braunii*

**DOI:** 10.1002/mbo3.482

**Published:** 2017-06-28

**Authors:** Christine Sambles, Karen Moore, Thomas M. Lux, Katy Jones, George R. Littlejohn, João D. Gouveia, Stephen J. Aves, David J. Studholme, Rob Lee, John Love

**Affiliations:** ^1^ Biosciences, College of Life and Environmental Sciences University of Exeter Exeter UK; ^2^ Present address: Bioprocess Engineering Group Wageningen UR AlgaePARC Wageningen The Netherlands

**Keywords:** biofuel, *Botryococcus braunii*, consortium, metagenomics, microcosm

## Abstract

Microalgae are widely viewed as a promising and sustainable source of renewable chemicals and biofuels. *Botryococcus braunii* synthesizes and secretes significant amounts of long‐chain (C_30_‐C_40_) hydrocarbons that can be subsequently converted into gasoline, diesel, and aviation fuel. *B. braunii* cultures are not axenic and the effects of co‐cultured microorganisms on *B. braunii* growth and hydrocarbon yield are important, but sometimes contradictory. To understand the composition of the *B. braunii* microbial consortium, we used high throughput Illumina sequencing of metagenomic DNA to profile the microbiota within a well established, stable B*. braunii* culture and characterized the demographic changes in the microcosm following modification to the culture conditions. DNA sequences attributed to *B. braunii* were present in equal quantities in all treatments, whereas sequences assigned to the associated microbial community were dramatically altered. Bacterial species least affected by treatments, and more robustly associated with the algal cells, included members of Rhizobiales, comprising *Bradyrhizobium* and *Methylobacterium*, and representatives of *Dyadobacter*,* Achromobacter* and *Asticcacaulis*. The presence of bacterial species identified by metagenomics was confirmed by additional 16S rDNA analysis of bacterial isolates. Our study demonstrates the advantages of high throughput sequencing and robust metagenomic analyses to define microcosms and further our understanding of microbial ecology.

## 1 INTRODUCTION

*Botryococcus braunii* (Trebouxiophyceae, Chlorophyta) is a green, colonial microalga found in fresh and brackish water habitats across the world (Metzger, Berkaloff, Casadevall, & Coute, 1985; Wolf, Nonomura, & Bassham, 2004). *Botryococcus braunii* characteristically produces copious amounts of C_30_‐C_40_ hydrocarbons and has therefore been proposed as a potential source of sustainable biofuel (Banerjee, Sharma, Chisti, & Banerjee, 2002; Chisti, 2007; Held et al., 1985; Hillen, Wake, & Warren, 1980; Metzger & Largeau, 2005). Algae may be cultured in open ponds or raceways, which are the most cost‐effective methods of culture for low value products such as biomass or biofuel (Richardson, Johnson, & Outlaw, 2012). However, highly selective conditions are required which favor hydrocarbon synthesis and maintain the dominance of the algal crop over other species.

The majority of *B. braunii* cultures are not axenic (Chirac, Casadevall, Largeau, & Metzger, 1985; Dayananda, Sarada, Usharani, Shamala, & Ravishankar, 2007; Metzger & Largeau, 2005; Wolf et al., 2004), but comprise a single algal strain and various associated microbes (Casadevall, Metzger, & Puech, 1984; Masters, 1971; Rivas, Vargas, & Riquelme, 2010; Wang & Xie, 1996) that co‐exist as a consortium. The interactions between microalgae and bacteria are complex: bacteria may stimulate algal growth by providing vitamins (Croft, Lawrence, Raux‐Deery, Warren, & Smith, 2005) and organic chelating agents (Amin et al., 2009), by releasing easily assimilated nitrogen derivatives (Paerl, 1977), by providing increased CO_2_, by generating inorganic nutrients, or by influencing the pH or redox potential (Jones, 1972; Mouget, Dakhama, Lavoie, & de la Noüe, 2006). Conversely, communities may include bacteria that restrict algal growth by competing for nutrients, degrading algal exopolysaccharides, releasing anti‐algal substances, or affecting algal morphology, reproductive structures and metabolic control (Banerjee et al., 2002). The addition of selected microorganisms to algal cultures, therefore, has the potential to increase stability and productivity. Nutrients from bacterial origin may provide an important strategy to maximize productivity. While the association with microorganisms is not essential for hydrocarbon production in *B. braunii*, the addition of single bacterial species to previously axenic *B. braunii* culture increased algal biomass and hydrocarbon yields (Chirac et al., 1985; Wang & Xie, 1996). However, these effects were variable and dependent not only on the bacterium present in the algal culture, but also on the algal strain and culture conditions (Chirac et al., 1985; Jones, 1972). Furthermore, the nature of the bacterial association with *B. braunii* was different when the algae were grown as a planktonic culture or in a biofilm (Rivas et al., 2010). It is clear, therefore, that microorganisms in the *B. braunii* consortium can have unpredictable and sometimes contrasting effects on algal biomass and hydrocarbon yield, which are essential considerations for sustainable biofuel production.

Metagenomic shotgun sequencing is a powerful alternative to rDNA sequencing for analyzing complex microbial communities (Cooper & Smith, 2015; Tringe & Rubin, 2005; von Mering et al., 2007). To investigate further the nature of the *B. braunii* microbial consortium, we profiled the microbiota of an established, laboratory culture of *B. braunii* under different conditions, using high throughput Illumina sequencing of metagenomic samples.

## 2 RESULTS AND DISCUSSION

### 2.1 Modification of *Botryococcus braunii* culture conditions

Understanding the structure of algal‐bacterial interactions using metagenomics offers the potential to ensure more robust cultures and exploit, for industrial or nutritional use, a wider variety of species than currently deployed. To investigate the nature of the *B. braunii* microbial consortium, and to discover which taxonomic units (species/genera) are present in close association with the alga, we used a combination of metagenomic analysis and identification of cultured isolates to investigate the bacterial species present in a laboratory culture of *B. braunii* strain Guadeloupe (Race B) (Metzger, David, & Casadevall, 1986).

**Figure 1:**
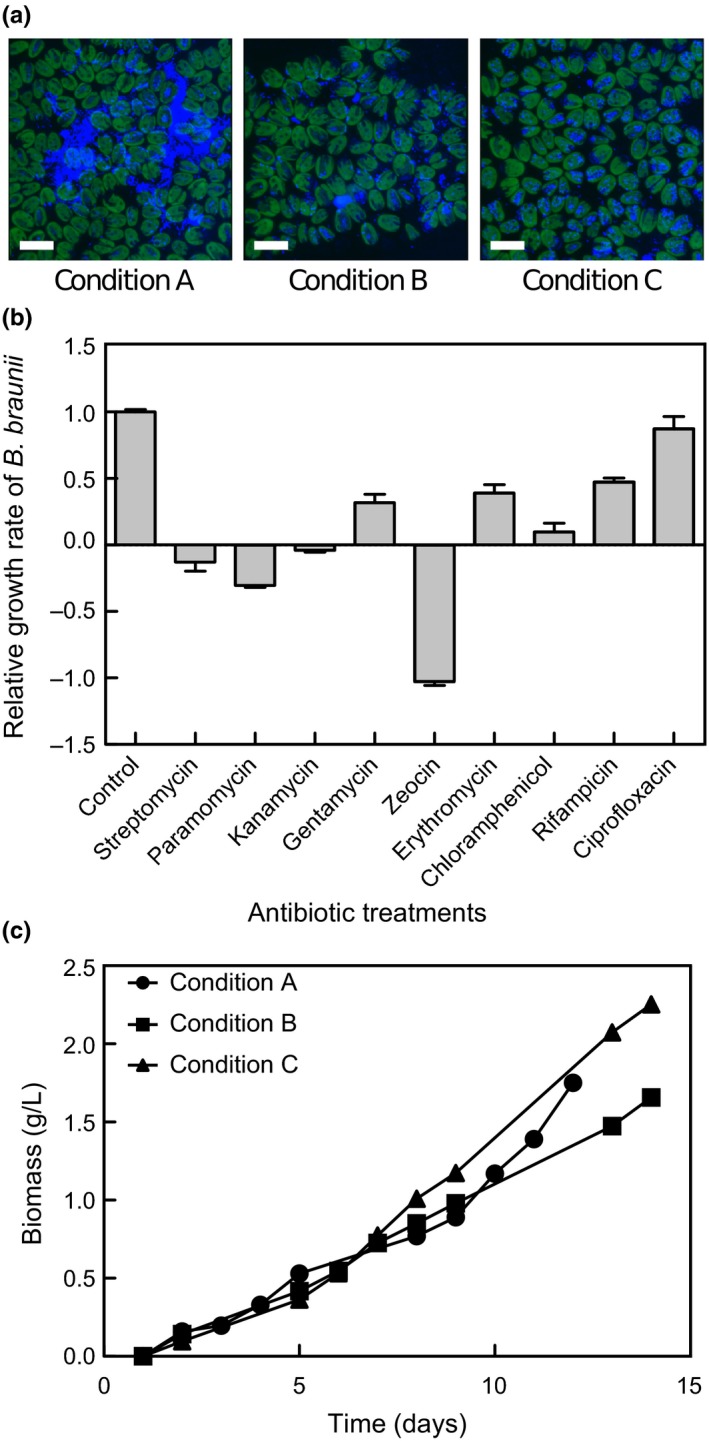
Response of the *Botryococcus braunii* (Guadeloupe) consortium to antibiotics. (a) DAPI staining of live cells of *B. braunii* (Guadeloupe) consortium. Scale bar = 20 μm. (b) *B. braunii* (Guadeloupe) consortium grown in MCV media supplemented with or without antibiotics. Algae growth was calculated by measuring chlorophyll extracted from culture; the growth rate was calculated from the change in chlorophyll concentration over 6 days (mg l^−1^  day^−1^) and divided by the average growth rate of the control (without antibiotics) to give the relative growth rate, so that control = 1; bars show the mean and SEM,* n* = 3. (c) Growth curves of *B. braunii* (Guadeloupe) consortium grown in MCV: initial consortium (Condition A: circle), washed culture (Condition B: square) and ciprofloxacin‐treated (Condition C: triangle). Biomass was calculated after drying algae at 60°C for 4–5 days. Data has baseline correction for different inocula and points represent the mean of three replicates

To drastically perturb the microbiota present in the *B. braunii* culture and identify bacteria that may be more closely associated with *B. braunii*, culture conditions were altered from those routinely used (Condition A) by either repeated centrifugation and rinsing (Condition B) or by addition of the antibiotic, ciprofloxacin (Condition C) to a laboratory culture of *B. braunii* (Figure 1).

Ciprofloxacin is a broad‐spectrum, fluoroquinolone antibiotic that inhibits DNA gyrase or topoisomerase IV activity (Maxwell, 1997) resulting in incomplete DNA synthesis during DNA replication and inhibition of bacterial growth. Ciprofloxacin was selected from a range of 10 antibiotics covering a range of activities and targets because it was the only antibiotic tested that had no significant effect on *B. braunii* growth and chlorophyll content (Figure 1).

**Figure 2:**
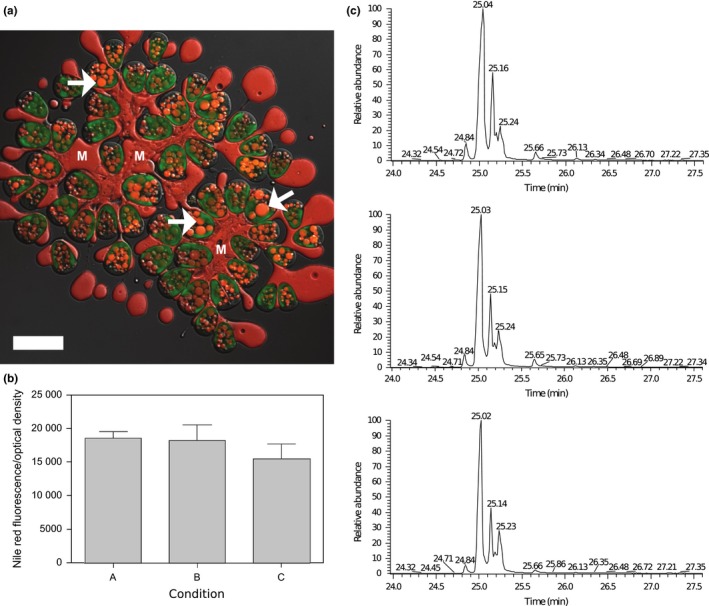
Effect of culture treatments on hydrocarbon production in *B. braunii*. (a) Lipid bodies and oil‐rich colony matrix (red) are distinguished from chloroplasts (green) in live cells of *B. braunii* using Nile red reagent. Images were captured using Zeiss LSM 510 META microscope (Carl Zeiss, Oberkochen, Germany) using a Plan‐Apochromat 63x/1.40 oil DIC M27 lens. Cells were irradiated with an excitation wavelength of 458 nm; emission of Nile red reagent was filtered between 550 and 571 nm whereas emission from chlorophyll was filtered between 668 and 721 nm. Individual cells are embedded within the oil‐rich matrix (M); lipid bodies are located within the cells (arrows). Scale bar = 20 μm. (b) Nile red fluorescence of *B. braunii* consortium 8 days after subculture; Nile red fluorescence was measured after incubating algal consortium with 3.2 μmol^–1^ Nile red reagent for 20 min and recording the fluorescence at an emission wavelength 560 nm after excitation at 490 nm. Fluorescence was calculated after removing the background fluorescence of the culture under identical conditions and Nile red fluorescence of the media control. Nile red fluorescence was normalized to the optical density of the consortium. Data are the mean and SEM of three replicates. (c) Representative chromatograms of the hydrocarbon fraction from *B. braunii* initial consortium (above), mechanically cleaned culture (middle) and ciprofloxacin‐treated cells (lower)

Nile red reagent is widely used to stain and quantify hydrocarbons produced by *B. braunii* ((Cooksey, Guckert, Williams, & Callis, 1987; Elsey, Jameson, Raleigh, & Cooney, 2007; Lee, Yoon, & Oh, 1998); Figure 2a). There was no significant difference in Nile red fluorescence (Figure 2b) or in the composition of hydrocarbons (Figure 2c) produced by *B. braunii* in conditions A, B or C. The observed changes in the microbiota between treatments therefore had no effect on the amount or composition of neutral lipids and hydrocarbons produced by the algae, which is consistent with previously reported data (Chirac et al., 1985). It is therefore likely that under all conditions tested the bacteria present were not having a negative impact on the growth or hydrocarbon metabolism of *B. braunii*.

### 2.2 Metagenomics analysis of the algal consortia

The development of high throughput metagenomic sequencing has enabled a profound examination of the complexity and dynamics of microbial populations (Cooper & Smith, 2015; Jansson, Neufeld, Moran, & Gilbert, 2012; Tringe & Rubin, 2005; von Mering et al., 2007). Metagenomic analysis can deliver information on the identity of microbes present in cultures and also data regarding the relative representation of different microbes within cultures.

Genomic DNA was extracted from all of the organisms present in conditions A, B and C and sequenced on the high‐throughput Illumina GA2 platform. After excluding low quality sequence reads, this analysis generated in excess of 11 million sequence reads per culture, namely 11,313,866 reads for condition A, 15,510,317 reads for condition B and 11,702,497 reads for condition C. The metagenomic content of the three conditions were analyzed using two bioinformatic approaches: 1. Similarity to nucleotide and protein databases using BLAST, and 2. Validation by sequence read alignment to genomes of inferred species identified using BLAST in step 1.

#### 2.2.1 Taxonomic assignment of metagenomic DNA using BLAST and MEGAN

To assess the range of bacteria at species level in all culture conditions, BLAST analysis was used to infer bacterial species present in the three experimental culture conditions. High quality sequence reads for Conditions A, B and C were analyzed by BLASTN using the NCBI nucleotide database (NT), and by BLASTX using the non‐redundant protein database (NR).

Using BLASTN to identify similar sequences and MEGAN to assign aligned reads to taxa, enabled taxonomic binning of 2,024,504 reads. The number of predicted bacterial species in the metagenome is dependent on the number of sequence reads that are binned to a given species.

High stringency analysis (100% sequence identity, 1,000 read threshold) identified 33 bacterial species in the metagenomes (Figure 4, Table S1). Standard stringency analysis (BLAST score ≥ 55; 10,000 read threshold) returned 22 bacterial species at a 10,000 read threshold (Table S2).

**Figure 3:**
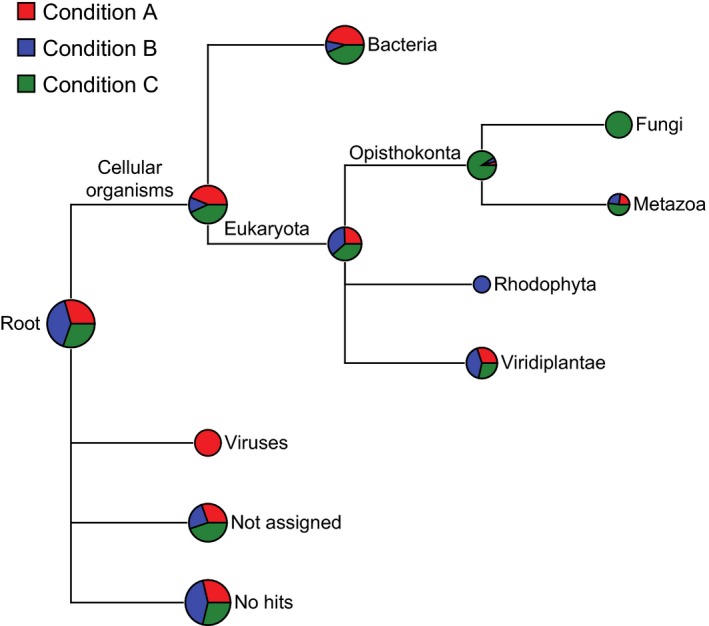
Distribution of taxa from the metagenome of *B. braunii*. BLAST was used to recognize similar sequences to each read in the NCBI nucleotide database and non‐redundant protein database. Taxa were assigned using MEGAN software at both standard and high stringency. The size of the circle is scaled logarithmically to the number of reads supporting the taxon; the proportion of the wedge indicates the numbers of reads from each culture condition: A (red), B (blue) and C (green)

Sequences of viral origin were found exclusively in the initial (Condition A) stock culture (Figure 3). These reads were almost entirely (99.8%) predicted to be from bacteriophages of the order Caudovirales. No sequences were binned to the Archaea. Sequences of eukaryotic origin aligned to Opisthokonta (Fungi and Metazoa), red algal (Rhodophyta) and plant (Viridiplantae) lineages (Figure 3). Reads binned to Viridiplantae (Figure 3) may be attributed to conserved *B. braunii* sequences and were predominantly assigned to *Vitis vinifera*,* Zea mays* and *Populus trichocarpa*, which could be ascribed to the abundance of nucleotide data present for these organisms in the BLAST databases. Bacterial assignments consisted of 83% of the binned metagenome (1,670,779 reads), which correlates to 4.34% of the entire metagenome.

The combined stringency analysis of BLASTX and BLASTN MEGAN assignment has identified species from 29 possible genera. As part of this investigation the high stringency and standard stringency analyses shared 19 identified species that may be present or similar to species which are present in the *B. braunii* consortium: *Achromobacter piechaudii*,* Acidovorax citrulli*,* Asticcacaulis excentricus*,* Bordetella petrii*,* Bradyrhizobium japonicum*,* Citrobacter koseri*,* Delftia acidovorans*,* Dyadobacter fermentans*,* Escherichia coli*,* Lactobacillus sakei*,* Lactococcus lactis*,* Leptothrix cholodnii*,* Methylibium petroleiphilum*,* Methylobacterium populi*,* Pseudomonas fluorescens*,* Salmonella enterica*,* Stenotrophomonas maltophilia*,* Variovorax paradoxus* and *Verminephrobacter eiseniae*.

#### 2.2.2 Further bacterial species resolution

High stringency MEGAN analysis was validated by alignments to previously sequenced genomes retrieved from NCBI of the identified species and the number and genome distribution of mapped reads determined. The number of reads from the entire metagenome that map to each of the 33 individual bacterial genomes identified by high stringency analysis were determined using Bowtie (Langmead, 2010; Langmead, Trapnell, Pop, & Salzberg, 2009).

To investigate the distribution and coverage of aligned reads across the genome, MOSAIK analysis revealed that none of the reference genomes was fully covered by Illumina reads (Table S4). The highest levels of coverage were for *Lactococcus lactis* (69.8%, condition A) and *Lactobacillus sakei* (48.1%, condition A). The reads were distributed evenly throughout the genomes rather than clustered together and resulted in a mean depth of coverage of 4.2× for *L. lactis* and 1.7× for *L. sakei*. This analysis confirms the presence of these or very closely related species in the microbiome.

**Figure 4:**
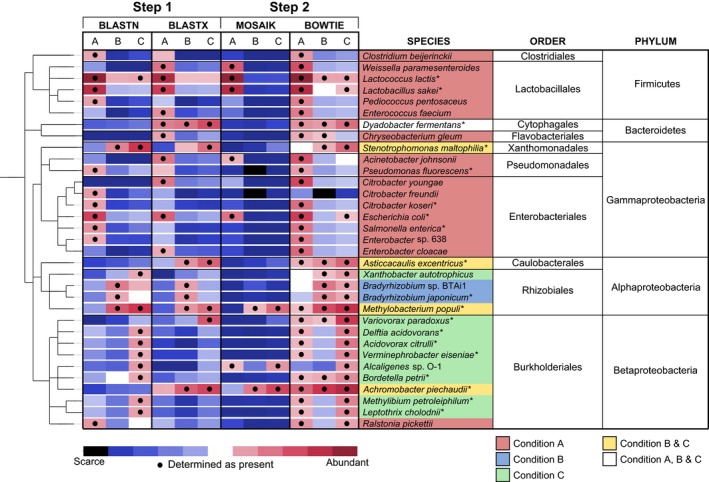
Taxonomic analysis of the *B. braunii* metagenome dataset. Step 1: Taxa were assigned using MEGAN restricted to bacteria with a heat map displaying abundance of reads assigned using BLASTN and BLASTX. Black dots represent likely presence based on high stringency cut‐offs; Step 2: Presence of taxa were identified using guided assembly of Illumina reads on to candidate genomes using Bowtie and Mosaik; mismatches were not allowed. Black dots represent likely presence based on cut‐offs: MOSAIK positive ≥ 8% coverage, BOWTIE positive ≥1500 reads. Stars represent species that were identified using both standard and high stringency analysis

The evidence from the combined high stringency analysis performed in this investigation indicated that the initial *B. braunii* culture (Condition A) contained a population of species from the orders Lactobacillales, Enterobacteriales, Pseudomonadales, Flavobacteriales, Clostridiales and one species in the Burkholderiales order, *Ralstonia pickettii* (Table S1, Figure 4). These microorganisms were removed by washing (Condition B) and failed to re‐colonize the culture when ciprofloxacin was present (Condition C). Species from the orders Cytophagales, Caulobacterales, Rhizobiales, Xanthomonadales and Burkholderiales (except *R. pickettii*) were not removed from the culture by washing (Condition B) suggesting that these species are very closely associated with, or attached to the algal colonies (Table S1, Figure 4).

Aside from two species, *Bradyrhizobium japonicum* and *Bradyrhizobium* sp. BTAi1, all identified Rhizobiales species were able to re‐colonize the culture at a lower level in the presence of ciprofloxacin (Condition C) (Table S1). Rinsing the culture resulted in an increase in alpha‐proteobacteria (mainly Rhizobiales), in particular, *B. japonicum* and *Bradyrhizobium* sp. BTAi1. Ciprofloxacin treatment reduced numbers of *Bradyrhizobium* spp. and *Enterobacter* sp. 638, which were superseded by *Methylobacterium*,* Xanthobacter*,* Asticcacaulis*,* Stenotrophomonas* and species from the order Burkholderiales, probably due to the creation of a suitable environmental niche.

The most striking aspect of this analysis is the presence of rhizobia that are commonly associated with endosymbiotic nitrogen fixation and are associated with roots of legumes and *Parasponia* (Cannabaceae). Rhizobia are a paraphyletic group that are found in the alpha‐ and beta‐proteobacteria. We have identified members of *Bradyrhizobium*,* Agrobacterium*,* Shinella, Xanthobacter and Methylobacterium* in the cultures either through metagenome or 16S sequencing of bacterial isolates. The rhizobial species identified through metagenomics appear to be fairly robustly associated with the algal colonies and are not removed by ciprofloxacin treatment. *Bradyrhizobium* are slow growing relative to other *rhizobia* and this has been reflected in the growth after washing and ciprofloxacin treatment. Co‐cultivation of *B. japonicum* has been shown to enhance the growth of *Chlamydomonas reinhardtii* (Wu, Li, Yu, & Wang, 2012).

#### 2.2.3 Validation of bioinformatics approaches by 16S rDNA sequencing of bacterial isolates

Following repeated streak‐culture on solid LB or MCV media, 10 bacterial strains with different colony characteristics (morphology, growth rate, medium preference) were isolated from the *B. braunii* consortium grown under Condition A. The genera of seven distinct bacterial strains were identified by 16S rDNA homology (Marchesi et al., 1998) as *Achromobacter*,* Asticcacaulis*,* Flavobacterium*,* Agrobacterium*,* Microbacterium*,* Shinella* and *Variovorax* (Table 1). Although similarity below 98.7%–99.0% of the 16S rDNA gene sequences of two bacterial strains has been reported as confirming they belong to different species (Stackebrandt & Ebers, 2006), the recent increase in metagenomic and bacterial sequencing has highlighted that 16S rDNA analysis can only refine identification to the family/genus level (Tu, He, & Zhou, 2014). All of these culturable genera, barring the *Microbacterium* and *Shinella*, were identified as present in the microcosm using metagenomic analysis, and their physical presence as isolated strains validates the accuracy of the methodology. These cultured isolates were subsequently sequenced and genomes assembled and published in 2016 where we reported the use of phylogenetic analysis and genome comparisons to further clarify, to at least genus level, five bacteria isolated from *B. braunii* as members of the *Achromobacter*,* Agrobacterium*,* Microbacterium* and *Shinella* genera (Jones et al., 2016).

**Table 1:** Identification of culturable bacteria in the *B. braunii* consortia

Species	Clone ID	Most similar strain	e‐value/identity	Accession	Match in metagenome
*Flavobacterium lindanitolerans*	GCSY	IP‐10	0/99%	NR_044208.1	*Flavobacterium johnsoniae* (SS1000)
*Microbacterium hydrocarbonoxydans* b	GCS4	BNP48	0/99%	NR_042263.1	NA
*Agrobacterium tumefaciens* a	SUL3	IAM 12048	0/99%	NR_041396.1	*Agrobacterium tumefaciens*/*vitis* (SS1000)
*Shinella granuli* a	GWS1 SUS2	Ch06	0/99%	NR_041239.1	NA
*Variovorax paradoxus* a	SUL4	S110	0/99%	NR_074654.1	*Variovorax paradoxus (HS)*
*Asticcacaulis excentricus* b	GCS3	CB 48	0/99%	NR_074137.1	*Asticcacaulis excentricus (HS)*
*Achromobacter spanius* b	GCS2	LMG 5911	0/99%	NR_025686.1	*Achromobacter piechaudii* (HS)

Most closely related species were identified by BLASTN of the 16S rDNA sequences from two independent clones with nucleotide identity of 99%. Top hits of species, clone ID and strain showing the highest similarity according to BLASTN with e‐value, percentage identity and NCBI accession number.

a Grew in fresh algal MCV media.

b Grew in spent but NOT fresh MCV media.

All bacterial isolates grew in LB medium. *Shinella*,* Agrobacterium* and *Variovorax* isolates also grew in fresh algal MCV medium at 25°C. *Achromobacter*,* Asticcacaulis*,* Microbacterium* and one unidentified colony (GG1) were unable to grow in fresh MCV but grew in “spent” MCV medium that was prepared from a filter‐sterilized culture of actively growing *B. braunii*. Moreover, *Shinella* showed improved growth in spent MCV compared to fresh MCV. The *Flavobacterium* failed to grow on fresh and spent MCV, suggesting that this strain requires another factor provided by the consortium to sustain growth.

Some bacterial species were unable to grow in MCV medium but were able to grow in filtered medium after *B. braunii* culture (unidentified species GG1, *Achromobacter* sp., *Asticcacaulis* sp. and *Microbacterium* sp.), suggesting they rely on factors produced by the algae or the other bacteria present in the algal consortium for growth. Further work in this area is required to determine which species in the consortium are beneficial and which are detrimental to *B. braunii*. *Rhizobium* species have been implicated as a probiotic in cultures of *B. braunii* (Rivas et al., 2010). In an attempt to understand how some of these interactions affect growth and hydrocarbon production, Chirac et al. (1985) investigated single species of bacteria combined with axenic cultures of *B. braunii*. Generally, biomass and hydrocarbon yield were increased compared to axenic cultures when CO_2_ was limiting, suggesting bacterially produced CO_2_ was utilized by the algae, but when CO_2_ was abundant both biomass and hydrocarbon yield were reduced. The precise effects depend on culture conditions, the *B. braunii* isolate and the microbial species involved (Chirac et al., 1985). The reasons for these potentially antagonistic or converse effects are complex and remain to be investigated fully. The presence of *Corynebacterium* sp. raised the level of hydrocarbon of the algal biomass (Banerjee et al., 2002) and *Bacillus* sp. increased hydrocarbon yield (Wang & Xie, 1996), however the precise reasons for the increases remain unknown. We have not observed any changes in hydrocarbon production when the consortia were perturbed, but as the growth rate was not significantly altered and the CO_2_ was not limiting, this is not necessarily unexpected.

Finally, to ascertain whether any of the isolated bacteria were resistant to ciprofloxacin, isolates were streaked on to LB‐agar containing 10 μg ml^−1^ ciprofloxacin. *Achromobacter*,* Flavobacterium* and *Variovorax* were resistant to the antibiotic, whereas *Asticcacaulis*,* Agrobacterium*,* Microbacterium* and *Shinella* were sensitive.

Some antibiotics resulted in significant changes in *B. braunii* growth rate and therefore may offer a means of altering the consortia in the future. The *B. braunii* consortium is not sensitive to antibiotics that inhibit peptidoglycan synthesis, whereas antibiotics that inhibit protein synthesis had a significant effect on the *B. braunii* consortium: streptomycin, kanamycin and paromomycin prevented growth of *B. braunii* resulting in decomposition of the algae; rifampicin, erythromycin, gentamycin and chloramphenicol reduce growth rate relative to the untreated culture without killing the algae.

**Figure 5:**
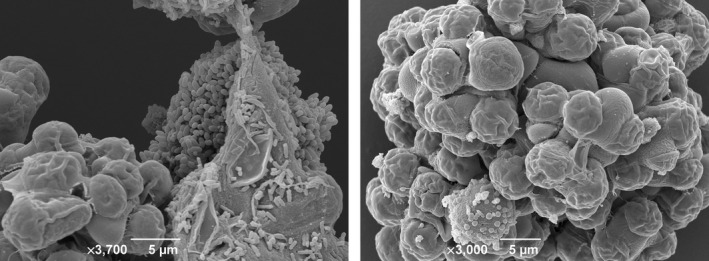
SEM images of *Botryococcus braunii* algae and associated bacteria. *Botryococcus braunii* consortia were imaged using cryogenic scanning electron microscopy. *B. braunii* cultures were washed with hexane to remove the hydrocarbons. Colonies were flash‐frozen in liquid N_2_ slush, transferred to a vacuum and coated in gold using the Gatan Alto 2100 system. Images were acquired using a JEOL JSM‐6390 LV scanning electron microscope at 5 kV with a working distance of 10–12 nm

The culturable bacteria species likely to be from the genera *Variovorax*,* Achromobacter* and *Flavobacterium* were resistant to ciprofloxacin (10 μg ml^−1^) whereas the *Agrobacterium* sp., *Shinella* sp., *Asticcacaulis* sp. and *Microbacterium* sp. were sensitive to ciprofloxacin when tested in isolation. An unexpected observation in this study was the sensitivity of the *Asticcacaulis* sp. to ciprofloxacin when grown on LB plates, but growth was evident in the consortia to a higher proportion than was present in the initial culture. This suggests that some additional factor was important for the increased presence of *Asticcacaulis* sp. in the consortia despite the ciprofloxacin, although it is possible that a very low level of ciprofloxacin‐resistant bacteria grew rapidly once the sensitive bacteria were inhibited or killed. An alternative possibility is that the *Asticcacaulis* sp. is protected in some way by the algae or perhaps these bacteria are attached to the *Botryococcus* extracellular matrix and the oil affords a barrier to the ciprofloxacin (Figure 5). *Asticcacaulis* sp. have been recorded in consortia with another Trebouxiophyceae alga, *Chlorella sorokiniana* (Watanabe et al., 2008) despite being a genus that is infrequently observed or isolated (Garrity et al., 2006). Another rarely observed species identified in this study was *Stenotrophomonas maltophilia*. It has been hypothesized to be a mutualistic bacterium when in culture with the dinoflagellate, *Scrippsiella trochoidea* (Tan et al., 2015).

## 3 CONCLUSIONS

The presence of co‐cultured bacteria is widely observed in laboratory cultures of microalgae and this may be associated with symbiotic associations that originate from the environment and persist in the laboratory. Studies on symbiotic associations of *C. sorokiniana* have revealed that microorganisms adhere to the surface of the algae directly or bind to the carbohydrate sheath produced by *Chlorella* providing the close proximity required for symbiotic association (Watanabe et al., 2005). Species from the genus *Methylobacterium* have been previously identified in *Chlorella vulgaris* and *C. sorokiniana* cultures (Guo & Tong, 2014; Watanabe et al., 2008). Co‐cultivation of *Methylobacterium radiotolerans* with *C. vulgaris* revealed the bacteria inhibited algal growth; this is in contrast to an isolated *Pseudomonas* species, which promoted algal growth under photoautotrophic conditions (Guo & Tong, 2014). Additionally, associations between *C. sorokiniana* and *Microbacterium* sp. significantly promoted growth of *C. sorokiniana* under photoautrophic conditions indicating mutualism (Watanabe et al., 2005). *Methylobacterium* sp. was identified in this metagenomics study and *Microbacterium* sp. was isolated from the *B. braunii* culture. *Microbacterium* sp. failed to grow in algal growth medium (MCV) but was able to grow in media after removal of the remaining *B. braunii* consortia by filtration, indicating that some factor produced by the consortium is required for maintenance of *Microbacterium* sp.

Other bacteria identified in this study have been previously observed in consortia with other green algae. *Dyadobacter fermentans* (Bacteroidetes) was present in all conditions in this study; *D. fermentans* has been identified in cultures of *C. sorokiniana* (Otsuka, Abe, Fukui, Nishiyama, & Senoo, 2008) and a *Dyadobacter* sp. has also been observed in culture with *C. vulgaris* (Lakaniemi, Hulatt, Wakeman, Thomas, & Puhakka, 2012). *D. fermentans* was originally isolated from the stems of *Zea mays* (Chelius & Triplett, 2000) and grows in nitrogen‐limited media with no currently known beneficial or detrimental effect on the host plant.

As well as species closely associated with *B. braunii* from the Bacteroidetes (e.g., *D. fermentans*,* S. maltophila*) and Alphaproteobacteria (e.g., *A. excentricus*,* M. populi*), several Betaproteobacteria (Burkholderiales) identified in this study have been observed in consortia with other green algae including *Variovorax* sp. (*C. vulgaris*), *Ralstonia* sp. (*C. vulgaris*,* C. sorokiniana*), *Acidovorax* sp. (*C. sorokiniana*), *Methylibium* sp. and *Leptothrix* sp. (*Cladophora glomerata*) (Lakaniemi et al., 2012; Otsuka et al., 2008; Watanabe et al., 2008; Zulkifly et al., 2012).

Investigation of a *Chlorella* consortium indicated that different bacteria utilized carbon and nitrogen compounds secreted by the algae in characteristic ways. This observation illustrates the complexity of interactions between the alga, the associated microcosm and the environment. When numerous species are present, the complexity of interactions is multiplied accordingly and the balances within the system are more likely to be perturbed. In order to culture *B. braunii* cost effectively at scale, understanding the composition of the community is essential.

Our results indicate that sequences attributed to the co‐cultured bacterial community were dramatically altered by the perturbations to the *B. braunii* culture. We reason that bacterial species least affected by the washing treatment (Condition B) were therefore more robustly associated with algal cells. These keystone species included members of Rhizobiales, comprising potentially symbiotic *Bradyrhizobium* and *Methylobacterium*, and representatives of the genera *Dyadobacter*,* Achromobacter* and *Asticcacaulis*.

These sequence data have been submitted to the NCBI SRA database under accession number SRP072490. Additional information on the methods used are available in Appendix S1.

## CONFLICT OF INTEREST

None declared.
