# Supplementary material for: Metagenomic analysis of the complex microbial consortium associated with cultures of the oil‐rich alga Botryococcus braunii
Source: Microbiologyopen. 2017 Jun 28;6(4):e00482. doi: 10.1002/mbo3.482 (PMC5552944; doi:10.1002/mbo3.482)
Supplement: Supplementary file 1 [file MBO3-6-na-s001.docx]

**Supp FIG 1**

Fig S1. The number of taxa assigned with varying number of supporting Illumina GAIIx reads in a single run. *B. braunii* cells from an initial population (Condition A: triangle) were compared to mechanically cleaned cells (Condition B: square) and ciprofloxacin-treated cells (Condition C: cross). A. Standard stringency and B. High stringency.

**Supp TABLE S1**

| **Species** | **BLAST NT** | **BLAST NR** | **MEGAN (NT)** | | | **MEGAN (NR)** | | |
| --- | --- | --- | --- | --- | --- | --- | --- | --- |
|  |  |  | **A** | **B** | **C** | **A** | **B** | **C** |
| *Achromobacter piechaudii* (NR) | 7 | 5,756 | 4 | 19 | 27 | 956 | 10,132 | 22,173 |
| *Acidovorax citrulli*^§^ (NT) | 10 | 4,640 | 125 | 30 | 2,096 | 4 | 0 | 18 |
| *Acinetobacter johnsonii** (NR) | 93 | 3,390 | 67 | 0 | 0 | 5,243 | 24 | 41 |
| *Alcaligenes* sp. O-1*** (NT) | 2 | 17 | 21 | 15 | 1,721 | 0 | 0 | 153 |
| *Asticcacaulis excentricus* (NR) | 4 | 3,840 | 5 | 9 | 31 | 233 | 2,106 | 9,219 |
| *Bordetella petrii*^§^ (NT) | 21 | 4,946 | 139 | 478 | 1,100 | 13 | 82 | 187 |
| *Bradyrhizobium japonicum*^§^ (NR/NT) | 726 | 9,030 | 45 | 1,144 | 447 | 19 | 1,840 | 308 |
| *Bradyrhizobium* sp. BTAi1^§^ (NR/NT) | 5 | 7,572 | 123 | 4,356 | 842 | 22 | 1,392 | 173 |
| *Chryseobacterium gleum** (NR) | 7 | 5,285 | 0 | 0 | 0 | 1,261 | 23 | 123 |
| *Citrobacter freundii** (NT) | 286 | 456 | 1,628 | 2 | 25 | 158 | 0 | 1 |
| *Citrobacter koseri*^§^ (NT) | 35 | 5,082 | 1,280 | 5 | 10 | 204 | 4 | 2 |
| *Citrobacter youngae** (NR) | 6 | 10,573 | 0 | 0 | 0 | 5,013 | 36 | 60 |
| *Clostridium beijerinckii**^§^ (NR/NT) | 43 | 5,082 | 1,157 | 1 | 7 | 663 | 0 | 0 |
| *Delftia acidovorans*^§^ (NT) | 94 | 6,149 | 278 | 36 | 1,686 | 19 | 3 | 142 |
| *Dyadobacter fermentans* (NR) | 7 | 5,703 | 13 | 17 | 29 | 3,978 | 5,566 | 18,495 |
| *Enterobacter cloacae** (NR) | 894 | 9,906 | 45 | 0 | 1 | 1,052 | 15 | 12 |
| *Enterobacter* sp. 638***^§^ (NT) | 3 | 4,247 | 1,033 | 7 | 6 | 295 | 1 | 4 |
| *Enterococcus faecium** (NR) | 809 | 26,451 | 240 | 0 | 3 | 2,334 | 7 | 26 |
| *Escherichia coli*^§^ (NR/NT) | 13,882 | 131,825 | 25,972 | 115 | 244 | 9,652 | 73 | 168 |
| *Lactobacillus sakei*^§^ (NR/NT) | 205 | 2,153 | 62,904 | 242 | 315 | 29,062 | 177 | 224 |
| *Lactococcus lactis*^§^ (NR/NT) | 1,866 | 10,684 | 225,243 | 949 | 1,146 | 91,000 | 547 | 608 |
| *Leptothrix cholodnii*^§^ (NT) | 3 | 4,345 | 228 | 32 | 1,486 | 15 | 2 | 32 |
| *Mesorhizobium loti*^†§^ (NT) | 618 | 7,766 | 39 | 70 | 295 | 7 | 17 | 149 |
| *Methylibium petroleiphilum*^§^ (NT) | 2 | 4,361 | 358 | 30 | 1,591 | 27 | 1 | 44 |
| *Methylobacterium populi*^§^ (NR/NT) | 9 | 5,326 | 108 | 12,896 | 35,006 | 13 | 2,036 | 6,253 |
| *Pediococcus pentosaceus**^§^ (NT) | 144 | 1,858 | 1,371 | 2 | 8 | 439 | 0 | 2 |
| *Polaromonas* sp. JS666^†§^ (NT) | 5 | 5,443 | 84 | 20 | 699 | 18 | 2 | 152 |
| *Pseudomonas fluorescens*^§^ (NR/NT) | 1,210 | 19,837 | 1,318 | 34 | 231 | 563 | 7 | 15 |
| *Ralstonia pickettii** (NT) | 99 | 8,771 | 1,799 | 53 | 482 | 258 | 19 | 97 |
| *Salmonella enterica*^§^ (NT) | 4,610 | 101,084 | 2,349 | 8 | 34 | 362 | 2 | 3 |
| *Stenotrophomonas maltophilia*^§^ (NR/NT) | 1,070 | 8,839 | 62 | 3,227 | 35,974 | 8 | 618 | 6,308 |
| *Variovorax paradoxus* (NR) | 108 | 6,307 | 0 | 4 | 226 | 19 | 158 | 13,988 |
| *Verminephrobacter eiseniae*^§^ (NT) | 13 | 4,916 | 68 | 15 | 1,044 | 4 | 4 | 222 |
| *Weissella paramesenteroides** (NR) | 36 | 1,977 | 90 | 0 | 0 | 5,801 | 20 | 37 |
| *Xanthobacter autotrophicus**^§^ (NT) | 23 | 5,011 | 9 | 207 | 1,084 | 0 | 45 | 211 |

**Table S1: High stringency analysis of *Botryococcus braunii* consortium meta-DNA**. Number of sequence reads assigned using MEGAN (MEGAN) identified from a BLAST (score = 55), 100% identity; *Absent from standard stringency analysis with a read cut-off of 10,000. † Present in standard stringency analysis with a read cut-off of 10,000 and absent from high stringency analysis. § Genome present in NT. (NT) denotes BLASTN analysis using nucleotide database; (NR) denotes BLASTX analysis using protein database; Consortium conditions: A, initial; B, washed; C, ciprofloxacin-treated consortia. Shading indicates presence.

**Supp TABLE S2**

| **Consortium species** | **BLASTN** | | | **BLASTX** | | | **Identification DB** |
| --- | --- | --- | --- | --- | --- | --- | --- |
|  | **A-NT** | **B-NT** | **C-NT** | **A-NR** | **B-NR** | **C-NR** |  |
| *Achromobacter piechaudii* | 1 | 4 | 6 | 1,088 | 11,768 | 27,702 | NR |
| *Acidovorax citrulli* | 1,047 | 397 | 27,113 | 4 | 0 | 104 | NT |
| *Asticcacaulis excentricus* | 6 | 12 | 53 | 756 | 6,741 | 27,824 | NT |
| *Bordetella petrii* | 907 | 4,338 | 10,997 | 40 | 123 | 348 | NR |
| *Bradyrhizobium japonicum* | 717 | 34,169 | 8,340 | 58 | 6,924 | 1,326 | NR |
| *Citrobacter koseri* | 19,490 | 116 | 151 | 184 | 1 | 6 | NT |
| *Delftia acidovorans* | 1,301 | 449 | 21,505 | 34 | 10 | 683 | NT |
| *Dyadobacter fermentans* | 19 | 13 | 31 | 15,981 | 24,551 | 84,657 | NT |
| *Escherichia coli* | 46,908 | 216 | 446 | 13,840 | 108 | 232 | NR |
| *Flavobacterium johnsoniae* | 1,015 | 744 | 14,535 | 365 | 386 | 6,801 | NT |
| *Lactobacillus sakei* | 134,192 | 554 | 690 | 40,755 | 243 | 317 | NR |
| *Lactococcus lactis* | 351,694 | 1,476 | 1,776 | 126,280 | 731 | 828 | NT |
| *Leptothrix cholodnii* | 1,956 | 203 | 11,822 | 128 | 17 | 146 | NT |
| *Mesorhizobium loti* | 530 | 2,918 | 13,799 | 8 | 142 | 786 | NT |
| *Methylibium petroleiphilum* | 2,018 | 187 | 13,926 | 121 | 5 | 281 | NR |
| *Methylobacterium populi* | 280 | 24,687 | 67,452 | 16 | 2,027 | 6,001 | NT |
| *Polaromonas sp. JS666* | 721 | 193 | 10,656 | 59 | 13 | 537 | NT |
| *Pseudomonas fluorescens* | 13,645 | 206 | 1,446 | 1,584 | 44 | 86 | NR |
| *Salmonella enterica* | 23,307 | 111 | 263 | 862 | 2 | 19 | NT |
| *Stenotrophomonas maltophilia* | 436 | 5,420 | 59,333 | 14 | 533 | 5,636 | NT |
| *Variovorax paradoxus* | 0 | 8 | 756 | 89 | 640 | 58,628 | NR |
| *Verminephrobacter eiseniae* | 507 | 339 | 23,130 | 11 | 23 | 1,387 | NT |

**Table S2: Standard stringency analysis of *Botryococcus braunii* consortium meta-DNA**. MEGAN identified 22 species with a cut-off of 10,000 reads. Consortium conditions: A, initial; B, washed; C, ciprofloxacin-treated consortia. (NT) denotes BLASTN analysis using nucleotide database; (NR) denotes BLASTX analysis using protein database; Taxa identified from BLAST with a minimum of 10,000 reads and a score of 55. Shading indicates presence.

**Supp TABLE S3**

| **Species (NCBI accession)** | **Bowtie (A)** | **Bowtie (B)** | **Bowtie (C)** |
| --- | --- | --- | --- |
| *Achromobacter piechaudii* ATCC 43553 *(*GCF_000164035.1*)* | 8,219 | 38,366 | 80,138 |
| *Acidovorax citrulli* AAC00-1 *(*GCF_000015325.1*)* | 748 | 251 | 5,066 |
| *Acinetobacter johnsonii* SH046 (GCF_000162055.1) | 13,014 | 245 | 476 |
| *Alcaligenes* sp. O-1 (AF109074) | 63 | 20 | 1,880 |
| *Asticcacaulis excentricus* CB 48 *(*GCF_000175215.2) | 855 | 2,285 | 7,972 |
| *Bordetella petrii* DSM 12804 *(*GCF_000067205.1*)* | 713 | 957 | 2,754 |
| *Bradyrhizobium japonicum / diazoefficiens* USDA 110 *(*GCF_000011365.1*)* | 419 | 2,665 | 1,080 |
| *Bradyrhizobium* sp. BTAi1 (GCF_000015165.1) | 472 | 4,907 | 1,265 |
| *Chryseobacterium gleum* ATCC 35910 (GCF_000143785.1) | 998 | 503 | 392 |
| *Citrobacter freundii* plasmid pCTX-M3 *(*AF550415.2*)* | 57 | 0 | 2 |
| *Citrobacter koseri* ATCC BAA-895 (GCF_000018045.1) | 6,383 | 191 | 340 |
| *Citrobacter youngae* ATCC 29220 (GCA_000155975.1) | 20,684 | 254 | 426 |
| *Clostridium beijerinckii* NCIMB 8052(GCF_000016965.1) | 1,850 | 55 | 111 |
| *Delftia acidovorans* SPH-1 (GCF_000018665.1) | 1,005 | 244 | 4,304 |
| *Dyadobacter fermentans* DSM 18053 (GCF_000023125.1) | 2,184 | 1,635 | 6,282 |
| *Enterobacter* sp. 638 (GCF_000016325.1) | 4,492 | 179 | 305 |
| *Enterobacter cloacae* ATCC 13047 (GCF_000025565.1) | 7,772 | 199 | 371 |
| *Enterococcus faecium* C68 (GCF_000160315.1) | 4,084 | 198 | 277 |
| *Escherichia coli* K-12 substr. MG1655 (GCF_000005845.1) | 27,201 | 284 | 522 |
| *Klebsiella pneumoniae* ATCC 700721; MGH 78578 (GCF_000016305.1) | 4,404 | 181 | 371 |
| *Lactococcus lactis* IL1403 (GCF_000006865.1) | 218,780 | 1192 | 1312 |
| *Lactobacillus sakei* 23K (GCF_000026065.1) | 75,646 | 468 | 605 |
| *Leptothrix cholodnii* SP-6 (GCF_000019785.1) | 861 | 217 | 2,985 |
| *Methylibium petroleiphilum* PM1 (GCF_000015725.1) | 1,017 | 236 | 3,392 |
| *Methylobacterium populi* BJ001 (GCF_000019945.1) | 528 | 17,636 | 45,234 |
| *Pediococcus pentosaceus* ATCC 25745 (GCF_000014505.1) | 3,579 | 183 | 248 |
| *Pseudomonas fluorescens* Pf0-1 (GCF_000012445.1) | 1,568 | 112 | 380 |
| *Ralstonia pickettii* 2D *(*GCF_000023425.1*)* | 1,991 | 327 | 1,236 |
| *Salmonella enterica* serovar Typhimurium str. LT2 *(*GCF_000006945.1) | 5,046 | 183 | 347 |
| *Stenotrophomonas maltophilia* K279a (GCF_000072485.1) | 458 | 857 | 7,817 |
| *Variovorax paradoxus* S110 *(*GCF_000023345.1*)* | 845 | 528 | 33,823 |
| *Verminephrobacter eiseniae* EF01-2 (GCF_000015565.1) | 603 | 211 | 2,888 |
| *Weissella paramesenteroides* ATCC 33313 *(*NZ_ACKU00000000*)* | 15,666 | 192 | 274 |
| *Xanthobacter autotrophicus* Py2 *(*GCF_000017645.1*)* | 443 | 576 | 1,551 |

**Table S3: Number of reads assigned to the genome using bowtie (BWT).**

Mismatches = 0; Shading indicates presence.

**Supp TABLE S4**

| **Species** | **Genome coverage (%) (A)** | **Genome coverage (%) (B)** | **Genome coverage (%) (C)** |
| --- | --- | --- | --- |
| *Achromobacter piechaudii* | 4.936 | 16.998 | 30.565 |
| *Acidovorax citrulli* | 0.031 | 0.008 | 0.176 |
| *Acinetobacter johnsonii* | 9.221 | 0.027 | 0.062 |
| *Alcaligenes sp. O-1* | 8.282 | 2.432 | 12.50 |
| *Asticcacaulis excentricus* | 0.173 | 0.613 | 1.645 |
| *Bordetella petrii* | 0.118 | 0.241 | 0.450 |
| *Bradyrhizobium japonicum* | 0.024 | 0.272 | 0.104 |
| *Bradyrhizobium* sp. BTAi1 | 0.055 | 1.202 | 0.306 |
| *Chryseobacterium gleum* | 0.260 | 0.008 | 0.015 |
| *Citrobacter freundii* | 0.095 | 0 | 0.067 |
| *Citrobacter koseri* | 1.185 | 0.002 | 0.005 |
| *Citrobacter youngae* | 5.021 | 0.017 | 0.042 |
| *Clostridium beijerinckii* | 0.735 | 0.001 | 0.002 |
| *Delftia acidovorans* | 0.092 | 0.006 | 0.096 |
| *Dyadobacter fermentans* | 0.418 | 0.286 | 0.861 |
| *Enterobacter sp. 638* | 1.019 | 0.003 | 0.006 |
| *Enterobacter cloacae* | 0.962 | 0.004 | 0.016 |
| *Enterococcus faecium* | 2.069 | 0.007 | 0.032 |
| *Escherichia coli* | 18.916 | 0.065 | 0.119 |
| *Klebsiella pneumoniae* | 0.606 | 0.005 | 0.031 |
| *Lactococcus lactis* | 69.835 | 1.100 | 1.133 |
| *Lactobacillus sakei* | 48.069 | 0.389 | 0.604 |
| *Leptothrix cholodnii* | 0.035 | 0.007 | 0.111 |
| *Methylibium petroleiphilum* | 0.221 | 0.299 | 0.176 |
| *Methylobacterium populi* | 0.082 | 8.714 | 20.524 |
| *Pediococcus pentosaceus* | 3.677 | 0.007 | 0.009 |
| *Pseudomonas fluorescens* | 0.232 | 0 | 0.005 |
| *Ralstonia pickettii* | 0.931 | 0.023 | 0.049 |
| *Salmonella enterica* | 0.807 | 0.001 | 0.007 |
| *Stenotrophomonas maltophilia* | 0.021 | 0.282 | 2.702 |
| *Variovorax paradoxus* | 0.042 | 0.058 | 1.817 |
| *Verminephrobacter eiseniae* | 0.014 | 0.002 | 0.063 |
| *Weissella paramesenteroides* | 24.485 | 0.062 | 0.124 |
| *Xanthobacter autotrophicus* | 0.002 | 0.115 | 0.369 |

**Table S4: Relative genome coverage was determined using Mosaik.** Shading indicates presence.

**Supp FIG 2**

**
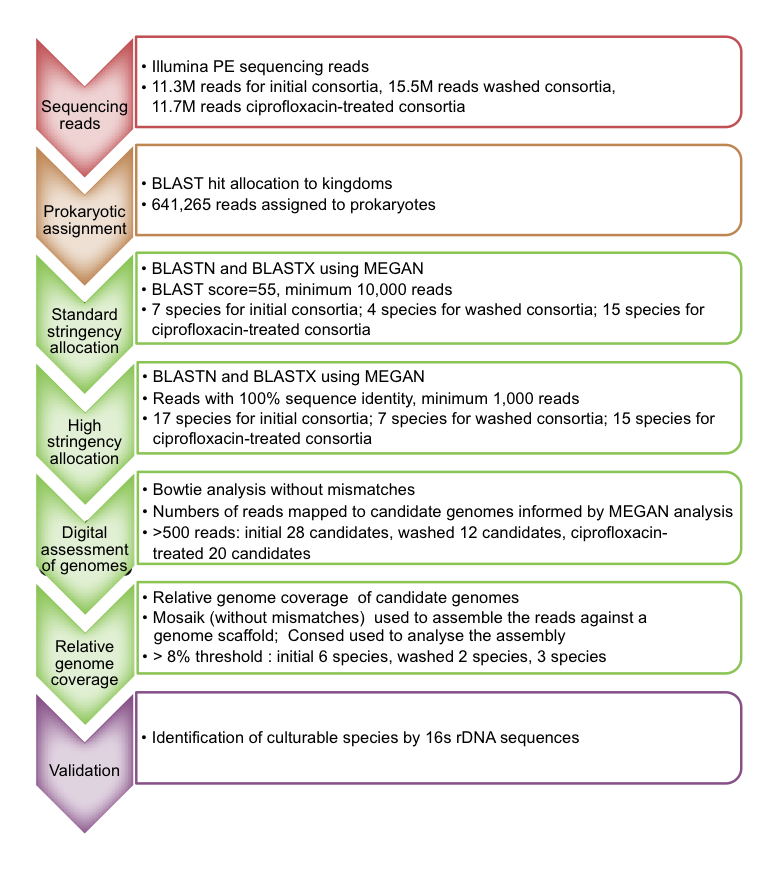
**

**Fig S2: Pipeline for metagenomic analysis of *B. braunii***
