# Supplementary material for: Metagenomic analysis of the complex microbial consortium associated with cultures of the oil‐rich alga Botryococcus braunii
Source: Microbiologyopen. 2017 Jun 28;6(4):e00482. doi: 10.1002/mbo3.482 (PMC5552944; doi:10.1002/mbo3.482)
Supplement: Supplementary file 2 [file MBO3-6-na-s002.docx]

# Experimental Procedures

### Botryococcus braunii culture

*Botryococcus braunii* race B, strain Guadeloupe (Metzger et al., 1986) was obtained from Pierre Metzger (Ecole Nationale Supérieure de Chimie de Paris, France) and has been continuously cultured in our laboratory since 2004. The algae and associated microbes (the *B. braunii* consortium) were cultured in 1 l conical flasks containing 200 ml of modified Chu13 medium supplemented with vitamins, citric acid and sodium selenate (MCV medium: 400 mg l^-1^ KNO_3_; 104.8 mg l^-1^ K_2_HPO_4_; 200 mg l^-1^ MgSO_4_.2H_2_O; 108 mg l^-1^ CaCl_2_.2H_2_O; 20 mg l^-1^ Fe-Na_2_EDTA; 9.4 µg l^-1^ Na_2_O_4_Se; 2.86 mg l^-1^ H_3_BO_3_; 1.8 mg l^-1^ MnCl_2_.4H_2_O; 220 µg l^-1^ ZnSO_4_.7H_2_O; 80 µg l^-1^ CuSO_4_.5H_2_O; 60 µg l^-1^ Na_2_MoO_4_.2H_2_O; 9 µg l^-1^ CoSO_4_.6H_2_O; 200 mg l^-1^ citric acid; 1.1 mg l^-1^ thiamine; 135 µg l^-1^ cobalamin; 25 µg l^-1^ biotin; 10 µl l^-1^ H_2_SO_4_; pH 7.2 with KOH), at 23 °C, in 5 % atmospheric CO_2_, under 100 µE m^-2^ s^-1^ white light (Sylvania Gro-Lux F15W/GRO fluorescent light) set to a 18 h:6 h light:dark cycle, with orbital shaking at 90 rpm. Flasks were inoculated with algae from a 5-day old, actively growing *B. braunii* seed culture such that the OD_680_ of new cultures was 0.2 (quantified using a Shimadzu UV-2101PC UV-VIS scanning spectrophotometer).

Algal growth was monitored by dry biomass and by the absorbance of extracted chlorophyll measured at 650 nm and at 665 nm, using a Shimadzu UV-2101PC UV-VIS scanning spectrophotometer. 10 ml aliquots of *B. braunii* cultures were filtered onto pre-weighed 2.7 µm GF/D glass-fibre membranes (Whatman) and either dried for 48 h at 60 °C or suspended in 10 ml methanol to extract the chlorophyll (Hipkins and Baker, 1986). Under these conditions chlorophyll concentration significantly correlated with biomass up to 2.5 g l^-1^.

### Physical and antibiotic treatments

The composition of the microbial consortium associated with *B. braunii* was investigated before (Condition A) and after rinsing (Condition B), and following antibiotic treatment (Condition C). For rinsing (Condition B), 200 ml of a 5 day-old *B. braunii* culture was centrifuged at 12,000 g for 15 min, resulting in a pellet composed largely of microorganisms and necrotic algal matrix, with the majority of algae remaining in the supernatant or floating at the surface. This supernatant was filtered through a 20 µm sieve. Small *B. braunii* colonies and unattached microorganisms were washed away with 10 volumes of sterile water. The algae retained by the sieve were transferred to fresh media to give an OD_680_ of 0.2 and cultured in 200 ml MCV for 14 days. For antibiotic treatments, cultures of rinsed algae were supplemented with 25 µg ml^-1^ chloramphenicol (Sigma, UK), 10 µg ml^-1^ ciprofloxacin (Fluka, UK), 50 µg ml^-1^ erythromycin (Sigma, UK), 50 µg ml^-1^ hygromycin B (Invitrogen, UK), 50 µg ml^-1^ kanamycin (Calbiochem, UK), 100 µg ml^-1^ paromomycin (Sigma, UK), 10 µg ml^-1^ streptomycin (Sigma, UK), 10 µg ml^-1^ rifampicin (Sigma, UK), 30 µg ml^-1^ gentamycin (Sigma, UK), or 5 µg ml^-1^ zeocin (Invitrogen, UK), immediately after transfer to fresh medium.

### Hydrocarbon quantification and analysis

Nile red (9-diethylamino-5H-benzo[α]phenoxazine-5-one; Sigma UK) was used for rapid determination of lipid production by *B. braunii* (Lee et al., 1998). 250 µl from *B. braunii* cultures were stained with 20 µl of 40 µM Nile red dissolved in acetone for 20 min. Nile red fluorescence was measured in a Tecan Infinite M-200 microplate reader, using an excitation wavelength of 490 ± 5 nm and an emission wavelength of 560 ± 20 nm. Cells were shaken for 5 s immediately before measurement. Analysis of hydrocarbons from selected samples was performed by gas chromatography / mass spectrophotometry (GC/MS). 25 mg of lyophilised algae (Scanvac coolsafe) were extracted in 17 ml HPLC grade hexane using a Dionex accelerated solvent extractor 150 (temperature 100 °C; static time 5 min; rinse volume 100 %; purge time 60 s; static cycle 2). The hexane extracts were then transferred to glass vials and purged with N_2_. Hydrocarbons and volatile compounds were separated and identified using a Trace GC/MS 2000 (Thermo Finnigan) equipped with an Rtx-5MS column (RESTEK, column dimensions: 15 m × 0.25 mm ID × 0.25 µm df (film thickness)). After splitless injection of the sample (1 µl) the GC oven was stabilised at an isothermal phase of 40 °C for 2 min and then heated at a rate of 10 °C min^-1^ to 320 °C which was held for 10 min. Injector temperature was maintained at 250 °C and the flow rate of the carrier gas (helium) was set to 1.0 ml min^-1^. The scan range of the mass spectrometer was from 30-700 m/z at a scan rate of 1.6 scans per second (ionisation energy: 70 eV).

### Metagenomic DNA purification and high throughput sequencing

Single samples of 200 ml from 14 day-old *B. braunii* cultures inoculated before rinsing (Condition A), after rinsing (Condition B) and after rinsing and treatment with 10 µg ml^-1^ ciprofloxacin (Condition C) were filtered through a glass-fibre C filter which retained both algae and some associated microorganisms. To ensure that all microorganisms were present in the meta-DNA sample, flow-through from the filter was centrifuged at 12,000 r.c.f. for 30 min and pelleted material added back to the filtered algae. Metagenomic DNA was purified using the plant DNeasy Maxi kit (Qiagen, UK). The plant DNeasy Maxi protocol included pulverising the material to in liquid N_2_ before adding buffer to the fine powder, thereby lysing both algae and bacteria, and providing a representative DNA sample of the entire algal / bacterial consortium.

Purified DNA was fragmented to below 1 kb using a Diagenode Bioruptor Sonicator UCD-200 (3 successive pulses of 15 min with 30 on / off cycles at medium intensity, on ice). Fragmented DNA was recovered using a QIAquick PCR purification kit (Qiagen, UK). DNA libraries were prepared for sequencing according to the Illumina protocol, using the Genomic DNA Sample Prep Kit (Illumina Inc.). Briefly, fragmented DNA was blunt ended using large Klenow fragment, T4 polynucleotide kinase and T4 polymerase, then an adenine residue was added to the 3’ terminus using Klenow exo-. Y-shaped paired-end adapters were ligated using DNA ligase and the engineered DNA library fragments separated by PAGE. Gel slices containing DNA libraries from 500-550 bp were excised and incubated overnight at 37 °C in 2 gel volumes of 0.3 M sodium acetate, 2 mM EDTA solution at pH 8.0. The DNA from the gel was precipitated, re-dissolved in 30 µl 10 mM Tris-HCl at pH 8.0, and 1 µl DNA was amplified by 18 PCR cycles. The quantity and quality of the libraries were verified using an Agilent Nanodrop 1000 Spectrophotometer, with an average concentration of 53.6 ng µl^-1^. Library size distribution was confirmed by electrophoresis in a 2 % agarose-TAE gel. Libraries were then diluted to 10 nM stock concentrations in 0.01 % TWEEN 20. Libraries were loaded on to an Illumina flowcell for cluster generation with v2 PE Cluster generation Kits on the Cluster Station (Illumina Inc.). A 76 bp paired sequencing run was performed on the Illumina Genome Analyzer II with v2.3 Sequencing Control Software (SCS) and Integrated Primary Analysis and Reporting (IPAR) using v3 Sequencing By Synthesis (SBS) reagents (Illumina, Inc.). Each library was sequenced in a separate lane with an average yield of 1.008 Tbp and an average of 85.23 % passing filter clusters.

### Bioinformatics metagenomics pipeline

Metagenomic DNA samples from each *B. braunii* culture (Conditions A, B and C) were sequenced using Illumina paired-end sequencing. The identification of species in the metagenomic DNA sample is based on the simultaneous alignment of sequenced fragments to sequences in nucleotide or protein databases within the constraint of their experimentally determined, spatial separation. We developed and tested a bioinformatics pipeline to analyse the sequence datasets for each *B. braunii* culture (Supplementary Figure 2).

First, BLAST (Altschul et al., 1990) was used to recognise similar sequences to each read in the NCBI nucleotide database (NT, containing 8,686,150 sequences) and non-redundant protein database (NR, containing 11,673,899 sequences). Taxa were assigned using MEGAN software (Huson et al., 2007) at both standard and high stringency. Paired-end information was retained when importing BLAST results into MEGAN.

Second, Bowtie software (Langmead et al., 2009; Langmead, 2010) quantified the number of reads mapped to specific genomes informed by high stringency MEGAN analysis.

Third, MOSAIK software (Lee et al., 2014) was used to assemble meta-DNA reads on to microbial genome reference sequences selected from the high stringency MEGAN output and based on the rationale that if a bacterium was indeed present in the consortium, its corresponding sequence reads would be evenly mapped across a major portion of the genome.

### Analysis of the high throughput metagenomic sequences from Botryococcus braunii consortia

The 76 bp paired end Illumina GAIIx sequencing reads obtained from a single flowcell lane were trimmed to 70 bp using FASTA/Q Trimmer (FASTX-Toolkit, Cold Spring Harbor Laboratory), based on sequence read quality for all samples. Sequences were excluded where over 4 % of bases had a quality of less than 20 (a common threshold score for high quality sequences) using FASTA/Q Trimmer (FASTX-Toolkit, Cold Spring Harbor Laboratory). Each of the retained 70 bp reads was analysed using BLASTN and BLASTX, either with a stringency of 100 % identity (high stringency) or with a cut-off score of 55 (standard stringency). The default filters were applied to remove low complexity regions. Taxa were assigned, rendered and compared (absolute counts) using the metagenomic analysis tool MEGAN (Huson et al., 2007) supplemented with paired end sequencing information. Parameters for MEGAN analysis were: min support 1000, min score 55, min score/length 0, top percent 10.0 and win score 0.

Confirmation of selected species was undertaken using Bowtie (Langmead et al., 2009; Langmead, 2010) to align reads to the genome sequence. A reference data set was generated for each genome using Bowtie; sequences and quality data were split and forward and reverse reads interwoven using the command line: (Bowtie -St -p 8 –n 0 <ref> <interweaved fastq reads> output.sam). Bowtie was run repeatedly using a different reference genome to determine aligned and unaligned reads.

As an alternative to MEGAN and Bowtie analysis, a reference-guided assembly of the Illumina sequences was performed against genomes from the species identified by high stringency MEGAN analysis. The reference-guided assembly was performed with MOSAIK (Lee et al., 2014) and the parameters chosen according the manual: -hs 15 -mm 0 -mhp 100 -act 20 -bw 29 -p8. Assemblies were analysed using consed (Gordon et al., 1998; Gordon, 2003; Gordon and Green, 2013) and the number of Illumina reads that comprised an assembly was quantified to confirm the presence of a particular bacterial strain in the *B. braunii* microbial consortium. Finally, to confirm the bioinformatics analysis,,bacterial isolates were cultured from an unperturbed *B. braunii* culture (Condition A) and identified by 16S rDNA sequencing.

### Isolation and identification of culturable bacteria in the initial Botryococcus braunii consortium

A single sample of 100 µl from a 14 day-old *B. braunii* culture was inoculated into fresh medium without rinsing (Condition A), diluted with 900 µl MCV and ten-fold serial dilutions performed to 10^-7^ of the original samples. 100 µl of each dilution plated on to MCV-1 % agar and LB-1 % agar plates and incubated at 25 °C for 2-7 days until bacterial colonies had formed. No evidence of *B. braunii* growth was observed on these plates. 10 different colony morphologies were observed and representative colonies were repeatedly streaked and cultured on the appropriate medium to isolate single strains. A single colony from each strain was cultured in 5 ml LB broth (10 g l^-1^ Bacto-tryptone, 5 g l^-1^ yeast extract, 10 g l^-1^ NaCl, pH 7.5) at 25 °C and DNA isolated from the bacteria using a Bacterial Genomic DNA Isolation Kit (Sigma, UK), according to the manufacturer’s instructions. The 16S rDNA sequence was amplified from each DNA sample using PCR. Reactions consisted of 1X Phusion HF buffer (NEB, UK), 8 mM dNTP mix (Promega, UK), 1 mM of prokaryotic 16S rDNA forward (63F; 5'-CAGGCCTAACACATGCAAGTC) and reverse (1387R; 5'-ACGGGCGGTGTGTACAAG) oligonucleotide primers (Marchesi et al., 1998), 1 unit Phusion thermostable DNA polymerase (NEB, UK) and 10 ng of purified bacterial DNA in 20 µl final volume. Initial denaturation of template DNA was at 98 °C for 30 sec, followed by 30 cycles consisting of 98 °C for 5 s, 55 °C for 20 s and 72 °C for 45 s, with a final incubation at 72 °C for 5 min. The amplified fragments were purified and cloned into pGEM-T (Promega, UK), sequenced by Beckman (UK), and aligned to the NCBI database using BLASTN.

***Scanning Electron Microscopy***

*Botryococcus braunii* consortia were imaged using cryogenic scanning electron microscopy. *B.braunii* cultures were washed with hexane to remove the hydrocarbons. Colonies were flash-frozen in liquid N_2_ slush, transferred to a vacuum and coated in gold using the Gatan Alto 2100 system. Images were acquired using a JEOL JSM-6390 LV scanning electron microscope at 5 kV with a working distance of 10 to 12 nm.
